# Maximizing the Reliability and Precision of Measures of Prefrontal Cortical Oxygenation Using Frequency-Domain Near-Infrared Spectroscopy

**DOI:** 10.3390/s24082630

**Published:** 2024-04-20

**Authors:** Elizabeth K. S. Fletcher, Joel S. Burma, Raelyn M. Javra, Kenzie B. Friesen, Carolyn A. Emery, Jeff F. Dunn, Jonathan D. Smirl

**Affiliations:** 1 Sport Injury Prevention Research Centre, Faculty of Kinesiology, University of Calgary, Calgary, AB T2N 4N1, Canada; raelyn.javra@ucalgary.ca (R.M.J.); kenzie.friesen@ucalgary.ca (K.B.F.); caemery@ucalgary.ca (C.A.E.); jonathan.smirl@ucalgary.ca (J.D.S.); 2 Hotchkiss Brain Institute, University of Calgary, Calgary, AB T2N 4N1, Canada; dunnj@ucalgary.ca; 3 Alberta Children’s Hospital Research Institute, University of Calgary, Calgary, AB T2N 4N1, Canada; 4 Libin Cardiovascular Institute of Alberta, University of Calgary, Calgary, AB T2N 4N1, Canada; 5 Integrated Concussion Research Program, University of Calgary, Calgary, AB T2N 4N1, Canada; 6 O’Brien Institute for Public Health, University of Calgary, Calgary, AB T2N 4N1, Canada; 7 Department of Radiology, Cumming School of Medicine, University of Calgary, Calgary, AB T2N 4N1, Canada; 8 Department of Clinical Neurosciences, Cumming School of Medicine, University of Calgary, Calgary, AB T2N 4N1, Canada

**Keywords:** near-infrared spectroscopy, reliability, brain oxygenation, cerebral blood flow, frequency-domain, methods

## Abstract

Frequency-domain near-infrared spectroscopy (FD-NIRS) has been used for non-invasive assessment of cortical oxygenation since the late 1990s. However, there is limited research demonstrating clinical validity and general reproducibility. To address this limitation, recording duration for adequate validity and within- and between-day reproducibility of prefrontal cortical oxygenation was evaluated. To assess validity, a reverse analysis of 10-min-long measurements (*n* = 52) at different recording durations (1–10-min) was quantified via coefficients of variation and Bland–Altman plots. To assess within- and between-day within-subject reproducibility, participants (*n* = 15) completed 2-min measurements twice a day (morning/afternoon) for five consecutive days. While 1-min recordings demonstrated sufficient validity for the assessment of oxygen saturation (S_t_O_2_) and total hemoglobin concentration (THb), recordings ≥4 min revealed greater clinical utility for oxy- (HbO) and deoxyhemoglobin (HHb) concentration. Females had lower S_t_O_2,_ THb, HbO, and HHb values than males, but variability was approximately equal between sexes. Intraclass correlation coefficients ranged from 0.50–0.96. The minimal detectable change for S_t_O_2_ was 1.15% (95% CI: 0.336–1.96%) and 3.12 µM for THb (95% CI: 0.915–5.33 µM) for females and 2.75% (95%CI: 0.807–4.70%) for S_t_O_2_ and 5.51 µM (95%CI: 1.62–9.42 µM) for THb in males. Overall, FD-NIRS demonstrated good levels of between-day reliability. These findings support the application of FD-NIRS in field-based settings and indicate a recording duration of 1 min allows for valid measures; however, data recordings of ≥4 min are recommended when feasible.

## 1. Introduction

At rest, the brain is one of the most perfused organs in the body, as adequate blood flow is critical for maintaining functionality [1]. This is due to the brain having minimal substrate storage, where individuals may experience syncope if blood flow is disrupted for as little as 8 to 12 seconds [2]. Cerebral blood flow disruptions occur in many acute and chronic brain injuries, and there are severe pathophysiological consequences of this form of injury [3]. The measurement of brain oxygenation currently largely relies on invasive techniques that carry higher risks of complications (e.g., cerebral perfusion pressure or jugular bulb oximetry) [4]. Having a reliable, quick, and non-invasive method for measuring cerebral oxygenation may aid brain monitoring in clinical and field-based settings, in addition to at-risk populations.

Many techniques have been employed to measure oxygenation as a biomarker for microvasculature function. A promising technique is a near-infrared spectroscopy (NIRS), of which there are three subtypes: continuous wave, time-domain, and frequency-domain [4]. In general, NIRS is a desirable imaging technique for its rapidity in assessment, portability, and non-invasive nature [5]. Frequency-domain NIRS (FD-NIRS) provides information with respect to the absolute concentrations of oxygenated and deoxygenated hemoglobin (HbO and HHb), whereas the most commonly used technique, continuous-wave NIRS, makes more assumptions about the photons’ pathlengths, and provides information only on relative changes in chromophore concentrations [6,7]. Further, compared with time-domain NIRS, FD-NIRS portability is similar but more cost-efficient, which is imperative for clinical utility [6,7,8].

To capitalize on the advantages of FD-NIRS and use the technique for field or bedside assessment of cerebral tissue oxygenation and hemoglobin content (often used as markers of perfusion), the reproducibility and reliability of the measure must be established. Current studies have shown FD-NIRS can reveal differences in perfusion between healthy controls and chronic conditions [9], but the distinction between healthy variation in cerebral oxygenation and variation due to disease state has yet to be established. Further, most validation and reliability studies have been limited to in vitro models and phantoms with known optical properties [10]. These models are not directly applicable to the adult head as this does not account for the heterogeneity in normal tissue and the various extracerebral layers (skin, skull, cerebral spinal fluid) [11,12,13]. For example, Davie and Grocott [14] show that three commonly used NIRS devices are all significantly contaminated by blood flow to the scalp tissue, and Dehaes et al. [15] used Monte Carlo simulations to show the influence of the thicker cerebrospinal fluid layer in newborns. One study of neonates established probe position–reposition reproducibility measures [16]; however, it is currently unknown if there are diurnal variations or daily variations resulting from natural physiological variability. 

If standards of variation are established, FD-NIRS could provide a valuable technique for monitoring changes in oxygen delivery in the brain without the risk of invasive techniques. There is currently no widely agreed-upon consensus regarding the optimal recording duration to obtain valid data to support the utility of FD-NIRS. Current standard practice involves performing 1-min-long recordings at rest and computing the mean value across the recording to provide a singular value for HbO, HHb, and THb [17]. Investigations of recording durations or trial numbers that are required to elicit valid results for other methods to assess cerebrovascular and cardiovascular function have been completed [18]; however, such analyses have not been completed with FD-NIRS. Currently, a lack of empirical evidence exists regarding the practice of taking the mean of a 1-min recording to produce valid assessments of prefrontal cortical oxygenation. The lack of a gold-standard approach for the quantification of microvascular circulation makes it difficult to be confident in the best practices for the minimal recording duration for FD-NIRS. This suggests that some problems in the reliability of the technique could potentially be associated with an inadequate recording duration and, thus, measurement error rather than true physiological changes. 

Therefore, the purpose of this study was to establish what recording duration is necessary to obtain valid measurements of prefrontal cortical oxygenation using FD-NIRS, in addition to determining the within- and between-day reliability of FD-NIRS-derived measures of prefrontal cortical oxygenation. Operationally, validity is quantified in this study by comparing different recording durations to a reference standard of 10 min, and reproducibility and reliability are quantified by comparing the similarity of oxygenation values within and between days. It was hypothesized that 1-min recordings may have moderately acceptable reproducibility; however, longer recording durations will have less variation in oxygenation measures and will display greater reproductivity [18]. It was additionally hypothesized that FD-NIRS measurements will have good within- and between-day reliability within subjects, with between-subject measurements will demonstrate more variability due to inter-individual differences in prefrontal cortical oxygenation [19].

## 2. Materials and Methods

The current study involved two investigations carried out to answer each of the two specific aims. The first aim was to establish the duration needed to maximize precision and efficiency in FD-NIRS recordings. The second aim was to establish the within- and between-day reliability of oxygenation measures determined by FD-NIRS. This project was approved by the University of Calgary Conjoint Health Research Ethics Board (REB20-2112). The experimental protocols and procedures used across studies were completed in congruence with all guidelines stated within the Declaration of Helsinki (revised version 2013, excluding the registration of the study) [20]. Before the commencement of this study, all protocols were explained thoroughly, the instrumentation was described, all questions were answered, and informed written consent was obtained from each participant.

Prefrontal cortical oxygenation measurements (S_t_O_2_, THb, HbO, and HHb) were obtained using a multi-distance (4 laser diodes with source–detector distances ranging from 2 to 4 cm), dual-wavelength (690 and 830 nm) frequency-domain near-infrared spectrometer (OxiplexTS, model 95205, ISS Inc., Champaign, IL, USA), with a sampling frequency of 2 Hz. Flexible forehead probes made of polyurethane with optical fibers embedded inside were used to obtain recordings of oxygenation measures. Prior to each recording, the probe was calibrated against a solid phantom of known optical properties, whose methods have been described extensively elsewhere [6]. The OxiplexTS device modulates the intensity of the lasers leaving the detector at a frequency of 110 MHz and measures the change in average intensity and phase shift of the detected light to determine the scattering and absorption coefficients of each wavelength of light and calculates the absolute concentrations of HbO and HHb in micro-moles per liter (µM) [12]. The FD-NIRS device was turned on to warm up and cycle the lasers for at least 30 min prior to every testing session.

All testing took place at the University of Calgary in a dark, quiet room, with the participants wearing noise-canceling headphones (Soundcore Model Q30, Anker Inc., Changsha, China) to block out any incidental noise. A trained researcher (E.K.S.F.) had the participants sit quietly in a comfortable chair while placing the flexible probe approximately 3 centimeters above the left eyebrow and 1 centimeter away from the midline of the forehead. There was a slight variance from these guidelines dependent on the individuals’ specific forehead anatomy. A small amount of clear ultrasound gel was used to ensure a good optical connection and minimize any potential influence of air between the forehead and the sensor array. The ultrasound gel was applied separately to the 4 source diodes and the detector to ensure there was no direct path for any photon to take directly between the sources and detector. The probe was secured with a cloth and dark headband to ensure that pressure was evenly applied and to limit the interference of ambient light. The researcher verified the values indicated strong signal quality with R^2^ > 0.85 on slope graphs comparing the source–detector distance to the alternating component of light intensity (linear slope relationship indicates ideal homogeneity of underlying tissue) before instructing the participant to close their eyes and remain relaxed during the testing. Recording took place once the participant was settled, and participants were only instructed to open their eyes after the recording was stopped to eliminate movement artifacts. Recordings were only completed on the left side of the forehead as specific hemispheric considerations have been shown not to impact FD-NIRS measurements at rest [21]. Participants were encouraged to relax as best as possible during the recordings and avoid complex thinking and executive functions (e.g., long-term planning and decision-making) [22]. Basic physiological parameters known to affect cerebral oxygenation (heart rate, blood pressure [BP], and partial pressure of end-tidal carbon dioxide [P_ET_CO_2_]) were collected in a separate session but under the same resting, quiet conditions due to equipment limitations. Heart rate was derived from the R-R interval collected using a 3-lead electrocardiogram. Beat-to-beat blood pressure was collected using finger photoplethysmography, with height corrected to the level of the heart (Finometer PRO, Finapres Medical Systems, Amsterdam, The Netherlands). P_ET_CO_2_ was collected with an inline gas analyzer and mouthpiece (ML206, AD Instruments, Colorado Springs, CO, USA) that was calibrated using a known gas concentration.

Data were collected from 52 healthy adults (26 females, 26 males; 26 ± 7 years) between July and September 2023. Of the 52 individuals who completed testing for the first aim (recording duration validity), 15 individuals (11 females, 4 males; 24 ± 4 years) returned to address the second aim (within- and between-day reproducibility). All participants recruited were healthy adults with no history of chronic neurological, musculoskeletal, respiratory, or cardiovascular conditions. 

To address the first aim of determining the optimal recording duration for FD-NIRS measurements, participants completed one testing session that lasted 10 min. After warmup and calibration of the FD-NIRS, each individual testing session consisted of the participant sitting at rest for a minimum of 5-min, positioning the probe to an area of strong optical connection, and an eyes-closed resting measurement for 10-min. Participants were allowed to keep their normal daily routine and habits as their data were going to be analyzed within-subject only and using only one recording (i.e., the mean value of 10-min would be compared with the mean of the first 9-min in the same person/session). This technique has been commonly used in other physiological datasets to assess the appropriate recording duration for other physiological measurements [18].

To assess within- and between-day reliability, fifteen participants returned twice a day for five consecutive days, once in the morning, 7–11 a.m., and once in the afternoon, 1–6 p.m., for ten sessions total. At least 4 h separated all visits, and each recording session lasted 2 min. To minimize confounding variables, participants were instructed to refrain from vigorous activity for 12 h before testing, refrain from nicotine and caffeine for 8 h, and maintain a similar routine for all days of testing.

Raw data were processed using customized MATLAB scripts (R2023b, 23.2.0.2391609). The OxiplexTS device samples at 2 Hz, giving 1200 data points for ten-minute recordings in objective 1. The mean HbO concentration, HHb concentration, THb concentration, and S_t_O_2_ were calculated for the entire duration as well as for the first 9- through 1-min recordings. This process was repeated for each individual recording session. For objective two, the same pre-processing system was used to find the mean of the blood metrics across each 2-min recording. 

All statistical analyses were performed using RStudio (2023.06.0421). For clarity, raw data presenting mean and standard deviations for the 1- through 10-min recording durations, as well as across the 10 time points for the within- and between-day reproducibility aim, are presented. Determination of optimal recording duration for FD-NIRS (i.e., validity) was conducted via (1) multivariable linear regression model with recording duration, sex, and history of concussion as predictors; (2) within-subject coefficient of variation (CoV); and (3) Bland–Altman plots with 95% limits of agreement (LOA). For the multivariable linear regression, it was predicted that a 1-min measurement would display larger variability; therefore, a large effect size was chosen at f^2^ = 0.35, while an alpha value of 0.05, power of 80%, and three predictor variables concluded a sample size of 36 was required. The within- and between-day reliability of FD-NIRS measurements were established via (1) repeated measure ANOVA, (2) within-subject CoV, (3) within-subject intra-class correlation coefficients (ICC), and (4) within-subject minimal detectable change (MDC) [23]. Based on an expected ICC of 0.875, a precision of 0.125 (good-to-excellent ICC range), an alpha of 0.05, and 10 repetitions, a sample of 8 participants were required [24]. 

The thresholds for CoV were established as <10% for good and <20% for reasonable/acceptable levels of variability, consistent with the cerebrovascular literature [25,26]. The thresholds for within-subject ICC were defined as <0.50 (poor), 0.50–0.75 (moderate), and 0.75–0.90 (excellent), in alignment with the existing literature [27].

## 3. Results

### 3.1. Demographics

Fifty-two participants completed testing to address objective 1. There was an even split of biological sexes for the participants (26 F, 26 M; no individuals reported as being non-cis-gendered; therefore, the interpretation of this study will focus solely on biological sex), and the median age was 24 years (IQR: 20.5–27.5 years). Nineteen of those assessed had a self-reported history of concussion (37%), and those with a self-reported concussion history had a median number of previous concussions of 2 (IQR: 0–2). Fifteen (11F, 4M) of the 52 participants completed testing to address objective 2, with a median age of 23 and an IQR of 6 years. Resting-state data on heart rate, blood pressure, and P_ET_CO_2_ were within normal ranges for young adults (Table 1).

### 3.2. Variability Based on Recording Duration

Across all recording durations, the mean values for oxygen saturation remained within a 0.5% range, the mean THb concentration remained within a 0.5 µM range with a constant downward trend as the duration of recording increased, and the mean HbO and HHb concentrations remained within a 0.22 and a 0.33 µM range, respectively (Table 2).

By using a multivariable linear regression model, no difference was found between any of the recording durations and the reference standard of 10 min (Table 3). There was a difference between females and males across all blood metrics (*p* < 0.001), with males having more THb, HbO, and HHb and higher S_t_O_2_ (Table 3). There was a difference in people who self-reported a history of at least one diagnosed concussion having slightly less HHb concentration (*p* = 0.049), but there were no other differences in S_t_O_2_, THb, or HHb (all *p* > 0.05) (Table 3).

For all recordings (1–9 min, inclusive), S_t_O_2_ and THb had a CoV less than 1%, demonstrating excellent validity with the 10-min recording (Table 4). S_t_O_2_ and THb displayed less variation as the recording duration increased (Table 4). Females and males had similar variation, with all of the confidence intervals associated with the CoVs overlapping between the sexes (Table 4); for HbO and HHb, a CoV of less than 1% occurred for recordings durations 4-min or longer (Table 4). Similarly, lower CoV for HbO and HHb were noted with a longer recording duration (Table 4).

**Figure 1:**
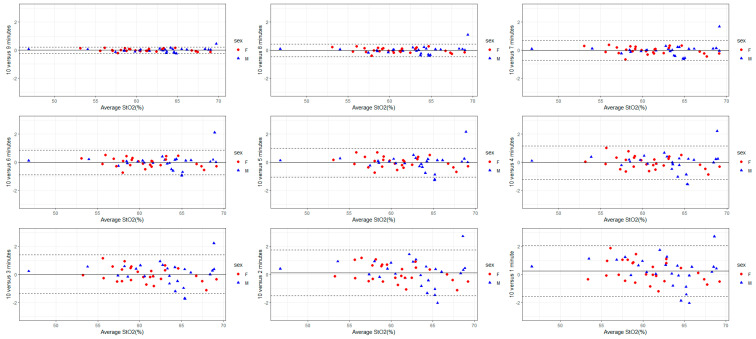
Bland–Altman plots with 95% LOA (dotted lines) demonstrate the validity of the interpretation of mean S_t_O_2_ values in reference to 10-min FD-NIRS recording durations. The means of shorter duration are compared with the mean of 10 min. The difference to the grand mean was ±2% in the 1-min recording and was reduced to ± <0.5% in the 9-min recording, indicating the decreased variability with longer recording durations.

**Figure 2:**
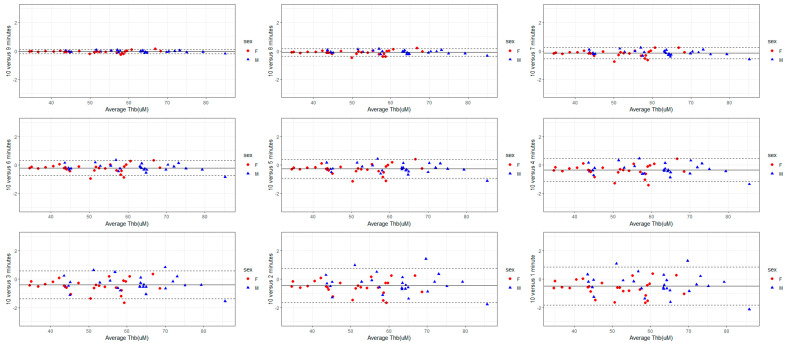
Bland–Altman plot with 95% LOA (dotted lines) comparing shorter lengths of FD-NIRS recordings in reference to 10-min-long recordings for THb concentration. Shorter recordings show a slight negative bias in the mean, and the difference at 1 min (±3 µM) was reduced to ± <0.5 µM with the 10-min recordings.

**Figure 3:**
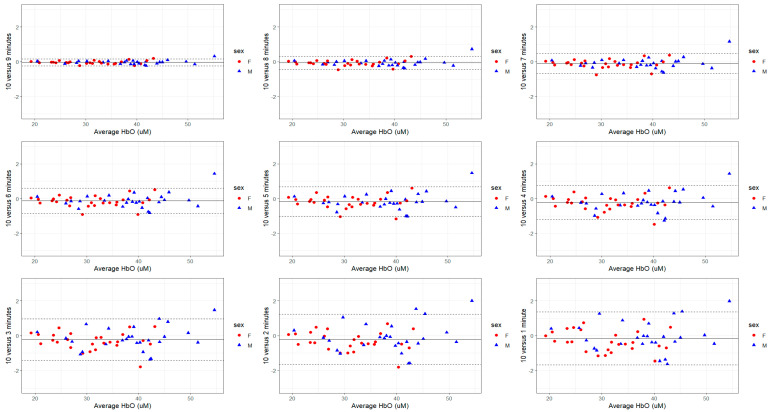
Bland–Altman plots with 95% LOA (dotted lines) comparing mean HbO concentration from shorter FD-NIRS recording durations to the reference standard of 10-min. The variability is maximized in the 1-min recordings with ±1.5 µM and minimized by the 9-min recording (±<0.05 µM).

**Figure 4:**
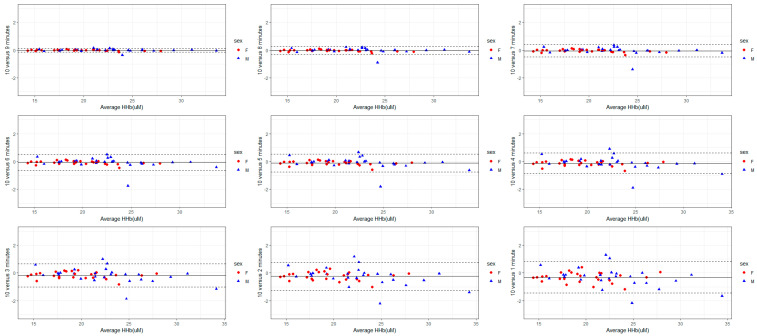
Bland–Altman plots with 95% LOA comparing shorter FD-NIRS recordings of HHb concentration to the reference standard of 10 min. The variability of HHb from 1 min recording (±1 µM) is halved by increasing the recording duration to 5 min (±0.5 µM).

The validity of each recording duration in reference to the mean S_t_O_2_, THb, HbO, and HHb derived from a 10-min-long measurement was analyzed using Bland–Altman plots (Figure 1, Figure 2, Figure 3 and Figure 4). The mean bias derived from one-minute-long measurements was approximately twice as large as the mean bias produced from a recording duration of 4-min and longer (Figure 1, Figure 2, Figure 3 and Figure 4). Nevertheless, the 95% LOA ranged from ±2% across all recording durations (Figure 1, Figure 2, Figure 3 and Figure 4). The same pattern exists for the other FD-NIRS-derived measures; as recording duration increases, less variability exists in the measure (Figure 1, Figure 2, Figure 3 and Figure 4). The THb concentration is the only measure that shows any bias in recording duration, with the shorter recordings tending to underestimate the THb concentration by 0.5 µM (Figure 2).

### 3.3. Within- and Between-Day Reproducibility

Analysis of within- and between-day reliability was completed using a repeated measure analysis of variance of S_t_O_2_ and THb, which revealed a main effect of time of day for both measures (F = 6.702, *p* = 0.021 for S_t_O_2_; F = 6.554, *p* = 0.023 for THb). Pairwise comparisons indicated morning measurements were different from afternoon measurements (t(74) = 2.778, *p* = 0.007 for S_t_O_2_; t(74) = 2.268, *p* = 0.026 for THb) (Table 5).

Using within-subject CoVs, FD-NIRS-derived measures of S_t_O_2_ and THb showed minimal variability, with CoVs smaller than 10%, even when comparing measurements across all time points (Table 6). Further, no difference was noted in the variability between males and females (Table 6). 

Using within-subject intraclass-correlation coefficients, the FD-NIRS measures of S_t_O_2_ and THb have good to excellent reliability across both sexes and all time points (Table 7). Females, in general, had better reproducibility than the males, particularly in the afternoon, where males had lower ICCs for both S_t_O_2_ and THb (0.50 and 0.69, respectively) (Table 7). The within-subject MDC for females was calculated to be 1.15% (95% CI: 0.336–1.96%) for S_t_O_2_ and 3.12 µM (95% CI: 0.915–5.33 µM) for THb. The within-subject MDC for males was 2.75% (95%CI: 0.807–4.70%) for S_t_O_2_ and 5.51 µM (95%CI: 1.62–9.42 µM) for THb.

## 4. Discussion

This study assessed the validity of different recording durations on various blood-based metrics assessed by FD-NIRS and subsequently assessed the within- and between-day reproducibility of a recording duration that optimized efficiency and reliability. The key findings were: (1) A 1-min recording duration had good reliability for mean S_t_O_2_ and THb concentration; however, recordings longer than 4-min may be more helpful for specific interpretations of mean HbO and HHb concentrations; (2) females have lower S_t_O_2_, THb, HbO, and HHb than males; (3) males and females have similar measurement variability and reproducibility; and (4) there may be diurnal variation in oxygenation measures where morning measurements appear to be slightly higher than in the afternoon.

For the time duration aim (aim 1), all recording durations had excellent consistency in mean S_t_O_2_ and THb (all CoV < 1%), with measures 4 min and longer also showing excellent consistency in mean HbO and HHb (Table 4). Across all time durations and blood-based metrics, females and males had similar variability (Table 2 and Table 4, Figure 1, Figure 2, Figure 3 and Figure 4). The linear model also showed a sex effect where females have lower S_t_O_2,_ THb, HbO, and HHb (Table 3). Females with lower THb, HbO, and HHb are supported in the literature, where females have lower blood volume [28] but higher cerebral blood flow [29,30]. This might suggest compensation for delivering a lower amount of oxygen (due to lower S_t_O_2_, hemoglobin concentrations, and lower blood volume) but at a higher rate, resulting in the same amount of oxygen delivery to the cerebral tissue [31]. Therefore, the finding of lower S_t_O_2_, THb, HbO, and HHb in females is unsurprising and makes mechanistic sense; however, why females had better reliability in FD-NIRS measurements is unknown. In general, longer recording durations yield less variability and allow for greater strength of analysis; however, for practical reasons, researchers will need to choose the duration associated with optimizing the validity of these data as well as minimizing participant burden. Due to the participant burden for the reproducibility aim (participants were required to attend 10 sessions, twice a day for 5 days, while also being restricted on caffeine and vigorous exercise), 2 min was chosen to optimize validity and time concerns. Based on the findings from the recording duration validity aim (aim 1), interpretation was limited to S_t_O_2_ and THb concentration. The repeated measure ANOVA and post-hoc tests revealed no difference between days but a significant effect of time of measurement within a day (morning versus afternoon) on both S_t_O_2_ and THb. Specifically, oxygen saturation and total hemoglobin content were slightly lower in the afternoon than in the morning (Table 5). In addition, within-subject CoV showed good reliability across time of measurement, sexes, and variables (Table 6), and all within-subject ICCs showed excellent reproducibility (Table 7). Using within-subject MDC, a difference of greater than 1.15% in S_t_O_2_ and 3.12 µM in THb for females and 2.75% in S_t_O_2_ and 5.51 µM in THb in males would indicate a significant change beyond the inherent resting variability.

To the researchers’ knowledge, there have been no previous investigations exploring the validity of collecting 1-min resting-state data and using the mean of these data points across the minute as the value for interpretation. Most research groups using FD-NIRS appear to either conduct real-time analysis to monitor changes in brain oxygenation in response to specific events (i.e., dynamic end-tidal forcing induced hypoxia [32], in neonates during the transition [33]), or they take the mean over 1 min of data collection (i.e., in neonates [17]). Depending on a study’s objectives, researchers have reported either optical coefficients, blood-based metrics, or both. However, based on the current results, reporting an estimation of HbO and HHb concentration by taking a mean for 1 min of data may introduce mild measurement bias, as the CoVs associated with this metric is >1% (Table 4). In addition, reporting total hemoglobin concentration from only 1 min of data might underestimate the true hemoglobin concentration as there was a negative bias with shorter recording durations found in this study (Figure 2).

Reproducibility studies of the FD-NIRS system have generally been limited to trials ex vivo using solid [6,34] or liquid phantoms [11,13]. Assessing reproducibility in this way assumes the human head is a homogenous material with consistent optical properties [34]; meanwhile, skin and skull contribute to light absorption [14], and the cerebrospinal fluid layer contains highly light-scattering membranes [35], making the generalizability across individuals’ (adolescent to adult, or between biological sexes) cerebral oxygenation severely limited. The between-day reproducibility from this investigation is supported by other research [21] that found good agreement with cross-correlation coefficients when elderly subjects returned for a second examination five months after the initial FD-NIRS measurement. Another study [36] reported similar results in large inter-subject variation, with a similar finding of approximately 6% variation in S_t_O_2_ in the brain, despite the mean values for S_t_O_2_ in this current study being approximately 10% higher than in the Choi et al. sample [36]. In addition, the Choi et al. investigation reported full sample variation; however, reproducibility was not achieved by failing to collect repeated measures on the same subjects [36]. Going beyond what the previous literature has investigated, this current study identified a potential diurnal effect on cerebral oxygenation; however, whether this is an interesting circadian rhythm finding or the effect of fatigue in individuals withheld from caffeine for the whole day is unable to be elucidated. Interestingly, this study found higher total hemoglobin concentration in both males and females than reported previously (Table 2 and Table 5). In a cadaveric-control brain oxygenation validation study, Gatto et al. reported their healthy controls (mean age 36 ± 9 years) had a THb concentration of 37.5 ± 8.6 µM [37], which is much lower than this study’s findings of 50.37 ± 9.59 µM for females and 61.08 ± 11.34 µM for males. Participants in this study were composed of a convenience sample of kinesiology students with higher-than-average cardiorespiratory fitness. Further, some individuals included were elite distance athletes; however, data on physical activity participation was not collected. It is unclear whether the discrepancy seen here is due to a potential training effect or differences in the probe itself.

The current study provides evidence for the reproducibility and utility of FD-NIRS in research and practical applications. Based on the findings outlined above, longer recording durations yield more precise measurements of prefrontal cortical oxygenation; however, recordings 1–2 min long still display good reliability and low variation (Table 3 and Table 4). For specific interpretation of HbO and HHb, a recording duration of 4 min or longer may provide a more reliable measure. In addition, within-subject comparisons should be performed wherever possible, and the significant sex effect on cerebral perfusion should be noted where females demonstrate lower S_t_O_2_, THb, HbO, and HHb levels (Table 3).

There are some limitations to this study that require mentioning. Despite coaching participants to relax, it is possible that subjects may have been breathing deeper than usual, potentially decreasing the partial pressure of carbon dioxide below eucapnic levels; however, current equipment limitations prevented concurrent monitoring of P_ET_CO_2_. In addition, this sample primarily included young adults, making the findings less generalizable to older adults or young children. This study aimed to address only biological sex effects, not gender differences, as there were only cis-gendered individuals who participated in this study, so further work with more focus on the social constructs associated with gender-diverse individuals should be undertaken in the future. Finally, there are known issues with NIRS systems failing to account for darker skin pigmentation and some loss of signal in individuals with more melanin pigmentation [38]. The interference of melanin with the quality of the NIRS signal is clearly a significant issue and may have contributed to some of the variation seen in this study, as over a third of the participants in the reproducibility trials self-identified as having darker skin. 

## 5. Conclusions

The purpose of this study was to establish the required recording duration to obtain valid measurements of prefrontal cortical oxygenation using FD-NIRS and to quantify the within- and between-day reliability of FD-NIRS-derived measures of prefrontal cortical oxygenation. It was observed that 1- and 2-min recordings had strong validity for interpretation of S_t_O_2_ and THb but recordings of 4-min or longer provided greater validity for HbO and HHb. Additionally, it was noted there was a large amount of inter-subject variability and a biological sex effect on mean S_t_O_2,_ THb, HbO, and HHb values at rest, highlighting the importance of comparing within-individuals rather than between. As such, whenever possible, it is recommended baseline recordings for the same individual are compared with their own follow-up data collections (e.g., for the concussion literature, ideally, collecting pre-injury baseline data with post-injury follow-ups may be more informative than comparing a post-injury data set to a control group). A limit for minimal change should be >1.15% in S_t_O_2_ and >3.12 µM in THb for females and >2.75% in S_t_O_2_ and >5.51 µM in THb for males. Due to the higher THb concentrations found in this study, caution may be needed when applying the MDC thresholds proposed in this study to participants of older age or with lower cardiorespiratory fitness who may have lower baseline THb concentrations. Consideration should be made for the time of day associated with FD-NIRS measurements, as there may be a diurnal effect on prefrontal cortical oxygenation at rest, with mornings yielding slightly higher results. Further research in FD-NIRS methodology should consider simultaneous monitoring of end-tidal gases and other physiological data, as well as considerations of the validity of measurement in individuals with darker skin pigmentation.

## Figures and Tables

**Table 1:** Basic physiological parameters, measured at rest for 10-min.

Physiological Parameter	Mean ± Standard Deviation	
	Female	Male
Heart rate (BPM)	70.3 ± 11.7	66.0 ± 9.20
Mean Blood Pressure (mmHg)	69.3 ± 8.59	81.4 ± 6.59
P_ET_CO_2_ (mmHg)	42.8 ± 6.81	35.6 ± 3.39

BPM beats per minute; mmHg millimeters of mercury; P_ET_CO_2_ partial pressure of end-tidal carbon dioxide.

**Table 2:** Mean and standard deviation of FD-NIRS derived blood metrics based on recording duration (*n* = 52, females = 26).

Duration (Minutes)	S_t_O_2_ (%)		THb (µM)		HbO (µM)		HHb (µM)	
	Female	Male	Female	Male	Female	Male	Female	Male
1	60.58 ± 4.20	62.34 ± 5.16	50.98 ± 9.65	61.44 ± 11.53	31.08 ± 7.08	38.44 ± 8.20	19.91 ± 3.50	23.00 ± 4.76
2	60.73 ± 4.17	62.46 ± 5.06	50.92 ± 9.64	61.42 ± 11.46	31.10 ± 7.06	38.49 ± 8.12	19.82 ± 3.51	22.92 ± 4.73
3	60.80 ± 4.13	62.57 ± 5.01	50.84 ± 9.61	61.41 ± 11.44	31.09 ± 7.03	38.56 ± 8.12	19.75 ± 3.51	22.85 ± 4.69
4	60.82 ± 4.09	62.64 ± 4.95	50.75 ± 9.60	61.39 ± 11.45	31.04 ± 7.00	38.59 ± 8.14	19.71 ± 3.50	22.80 ± 4.65
5	60.81 ± 4.05	62.65 ± 4.93	50.67 ± 9.58	61.33 ± 11.43	30.98 ± 6.96	38.56 ± 8.17	19.69 ± 3.51	22.77 ± 4.60
6	60.82 ± 4.02	62.63 ± 4.90	50.60 ± 9.58	61.27 ± 11.42	30.94 ± 6.95	38.52 ± 8.17	19.66 ± 3.52	22.75 ± 4.56
7	60.82 ± 4.01	62.62 ± 4.88	50.54 ± 9.58	61.21 ± 11.41	30.90 ± 6.95	38.48 ± 8.21	19.64 ± 3.52	22.73 ± 4.51
8	60.81 ± 3.97	62.62 ± 4.90	50.48 ± 9.58	61.17 ± 11.39	30.86 ± 6.94	38.46 ± 8.23	19.62 ± 3.51	22.70 ± 4.48
9	60.79 ± 3.96	62.62 ± 4.92	50.42 ± 9.59	61.12 ± 11.37	30.82 ± 6.95	38.44 ± 8.26	19.60 ± 3.50	22.68 ± 4.46
10	60.79 ± 3.93	62.60 ± 4.95	50.37 ± 9.59	61.08 ± 11.34	30.79 ± 6.95	38.40 ± 8.28	19.58 ± 3.48	22.67 ± 4.45

S_t_O_2_ oxygen saturation; THb total hemoglobin concentration; HbO oxyhemoglobin concentration; HHb deoxyhemoglobin concentration.

**Table 3:** Linear model comparing different recording durations of FD-NIRS to a reference 10-min, with sex and concussion history as predictor variables. Total sample *n* = 52, 26 females.

Variables	S_t_O_2_	THb	HbO	HHb
9 min	0.2 (95%CI: −1.5, 2.0) *p* = 0.790	−0.5 (95%CI: −4.5, 3.5) *p* = 0.811	−0.2 (95%CI: 3.1, 2.7) *p* = 0.912	−0.3 (95% CI: −1.9, 1.2) *p* = 0.679
8 min	0.1 (95% CI: −1.6, 1.9) *p* = 0.879	0.0 (95% CI: −4.1, 4.0) *p* = 0.982	(95% CI: −2.9, 2.9) *p* = 0.979	−0.1 (95% CI: −1.6, 1.5) *p* = 0.915
7 min	0.2 (95% CI: −1.5, 2.0) *p* = 0.795	−0.1 (95% CI: −4.1, 3.9) *p* = 0.965	0.1 (95% CI: −2.8, 3.0) *p* = 0.964	−0.2 (95% CI: −1.7, 1.4) *p* = 0.845
6 min	0.3 (95% CI: −1.5, 2.0) *p* = 0.756	−0.1 (95% CI: −4.2, 3.9) *p* = 0.945	0.1 (95% CI: −2.8, 3.0) *p* = 0.968	−0.2 (95% CI: −1.8, 1.4) *p* = 0.799
5 min	0.3 (95% CI: −1.5, 2.0) *p* = 0.760	−0.2 (95% CI: −4.2, 3.8) *p* = 0.917	0.0 (95% CI: −2.9, 2.9) *p* = 0.992	−0.2 (95% CI: −1.8, 1.3) *p* = 0.774
4 min	0.3 (95% CI: −1.5, 2.0) *p* = 0.766	−0.3 (95% CI: −4.3, 3.7) *p* = 0.891	−0.0 (95% CI: −2.9, 2.9) *p* = 0.984	−0.3 (95% CI: −1.8, 1.3) *p* = 0.752
3 min	0.3 (95% CI: −1.5, 2.0) *p* = 0.770	−0.3 (95% CI: −4.4, 3.7) *p* = 0.868	−0.1 (95% CI: −3.0, 2.8) *p* = 0.965	−0.3 (95% CI: −1.8, 1.3) *p* = 0.730
2 min	0.3 (95% CI: −1.5, 2.0) *p* = 0.772	−0.4 (95% CI: −4.4, 3.6) *p* = 0.848	−0.1 (95% CI: −3.0, 2.8) *p* = 0.948	−0.3 (95% CI: −1.9, 1.3) *p* = 0.709
1 min	0.2 (95% CI: −1.5, 2.0) *p* = 0.781	−0.4 (95% CI: −4.5, 3.6) *p* = 0.829	−0.1 (95% CI: −3.0, 2.8) *p* = 0.930	−0.3 (95% CI: −1.9, 1.2) *p* = 0.693
Sex	**1.8 (95% CI: 1.0, 2.5)** ***p* < 0.001**	**11 (95% CI: 8.9, 12)** ***p* < 0.001**	**7.6 (95% CI: 6.3, 8.9)** ***p* < 0.001**	**3.1 (95% CI: 2.4, 3.8)** ***p* < 0.001**
Concussion history	0.8 (95% CI: −0.0, 1.6) *p* = 0.060	−1.5 (95% CI: −3.3, 0.4) *p* = 0.125	−0.7 (95% CI: −2.1, 0.6) *p* = 0.283	**−0.7 (95% CI: −1.4, −0.0)** ***p* = 0.049**

S_t_O_2_ oxygen saturation; THb total hemoglobin concentration; HbO oxyhemoglobin concentration; HHb deoxyhemoglobin concentration.

**Table 4:** Within-subject coefficients of variation for each FD-NIRS-derived blood metric according to recording duration, with 10-min as the reference. Total sample = 52 (26 females).

Duration (Minutes)	S_t_O_2_ (% [95% CI])		THb (% [95% CI])		HbO (% [95% CI])		HHb (% [95% CI])	
	Female	Male	Female	Male	Female	Male	Female	Male
1	0.76 (0.53, 1.00)	0.94 (0.63, 1.25)	0.96 (0.70, 1.21)	0.75 (0.52, 0.99)	1.32 (1.01, 1.62)	1.27 (0.88, 1.65)	1.39 (0.96, 1.82)	1.77 (1.16, 2.37)
2	0.65 (0.49, 0.81)	0.83 (0.54, 1.11)	0.85 (0.62, 1.08)	0.72 (0.52, 0.93)	1.14 (0.82, 1.45)	1.22 (0.85, 1.59)	1.08 (0.74, 1.41)	1.46 (0.88, 2.04)
3	0.54 (0.40, 0.68)	0.67 (0.43, 0.91)	0.75 (0.54, 0.95)	0.61 (0.46, 0.77)	1.01 (0.70, 1.32)	1.03 (0.72, 1.33)	0.82 (0.55, 1.09)	1.18 (0.69, 1.67)
4	0.45 (0.32, 0.57)	0.51 (0.27, 0.75)	0.62 (0.43, 0.80)	0.49 (0.38, 0.61)	0.84 (0.55, 1.12)	0.85 (0.60, 1.11)	0.62 (0.40, 0.85)	0.97 (0.50, 1.45)
5	0.38 (0.28, 0.48)	0.42 (0.20, 0.63)	0.51 (0.35, 0.66)	0.39 (0.30, 0.49)	0.71 (0.47, 0.95)	0.72 (0.50, 0.93)	0.50 (0.32, 0.68)	0.78 (0.35, 1.22)
6	0.32 (0.24, 0.41)	0.35 (0.16, 0.54)	0.40 (0.28, 0.52)	0.30 (0.23, 0.37)	0.59 (0.38, 0.79)	0.57 (0.38, 0.76)	0.39 (0.25, 0.53)	0.66 (0.25, 1.06)
7	0.25 (0.18, 0.32)	0.27 (0.12, 0.41)	0.29 (0.19, 0.39)	0.21 (0.16, 0.26)	0.45 (0.29, 0.62)	0.42 (0.27, 0.56)	0.29 (0.18, 0.40)	0.50 (0.18, 0.81)
8	0.17 (0.13, 0.21)	0.18 (0.09, 0.28)	0.19 (0.13, 0.26)	0.14 (0.10, 0.17)	0.32 (0.22, 0.42)	0.27 (0.18, 0.36)	0.19 (0.12, 0.26)	0.33 (0.12, 0.54)
9	0.09 (0.07, 0.12)	0.11 (0.07, 0.15)	0.10 (0.07, 0.14)	0.07 (0.05, 0.09)	0.18 (0.13, 0.23)	0.15 (0.10, 0.20)	0.12 (0.07, 0.16)	0.18 (0.09, 0.27)

S_t_O_2_ oxygen saturation; THb total hemoglobin concentration; HbO oxyhemoglobin concentration; HHb deoxyhemoglobin concentration.

**Table 5:** Means and standard deviations * for FD-NIRS-derived blood oxygenation metrics, indicating average resting values and variability across five consecutive days. The total sample included 15 individuals (11 females). Morning measurements were collected between 7–11 am, and afternoon measurements were collected between 1–6 pm. Within-subject measurements were separated by at least 4 h.

Time of Measurement	Oxygen Saturation (%)		Total Hemoglobin Concentration (µM)	
	Female	Male	Female	Male
Morning	60.58 ± 0.95	61.80 ± 0.62	52.53 ± 2.75	62.06 ± 2.82
Afternoon	60.09 ± 0.67	59.21 ± 0.43	51.45 ± 2.37	58.62 ± 3.65
Combined	60.34 ± 0.58	60.51 ± 0.54	51.98 ± 2.17	60.34 ± 2.50

* Within-subject means and standard deviations were calculated and then averaged between subjects.

**Table 6:** Within-subject coefficient of variation comparing the amount of variability in FD-NIRS derived blood oxygenation measures in males and females across different time points and 5 consecutive days, *n* = 15 (11 females). Morning measurements were collected between 7–11 a.m., and afternoon measurements were collected between 1–6 p.m. Within-subject measurements were separated by at least 4 h.

	S_t_O_2_ (% [95% CI])		THb (% [95% CI])	
	Female	Male	Female	Male
Morning	3.60 (2.61–4.60)	2.42 (0.90–3.93)	9.42 (5.55–13.3)	5.99 (0.00–12.6)
Afternoon	3.31 (2.54–4.08)	5.34 (4.14–6.54)	6.03 (2.90–9.16)	10.2 (0.00–20.9)
Combined	3.55 (2.93–4.18)	4.55 (3.27–5.82)	8.27 (5.05–11.5)	8.96 (2.10–15.8)

S_t_O_2_ oxygen saturation; THb total hemoglobin concentration.

**Table 7:** Within-subject intraclass correlation coefficients assess the reliability of oxygen saturation and total hemoglobin concentration measurements. The total sample included 15 individuals (11 females). Morning measurements were collected between 7–11 a.m., and afternoon measurements were collected between 1–6 p.m. Within-subject measurements were separated by at least 4 h.

	S_t_O_2_ (ICC, [95% CI], *p*-Value)		THb (ICC, [95% CI], *p*-Value)	
	Female	Male	Female	Male
Morning	0.92, (0.811–0.975) *p* < 0.001	0.94 (0.74–1.00) *p* < 0.001	0.76 (0.44–0.93) *p* < 0.001	0.83 (0.18–0.99) *p* = 0.01
Afternoon	0.94 (0.85–0.98) *p* < 0.001	0.50 (0.00– 0.97) *p* = 0.18	0.92 (0.81–0.98) *p* < 0.001	0.69 (0.00–0.98) *p* = 0.05
Combined	0.96 (0.92–0.99) *p* < 0.001	0.87 (0.56–0.99) *p* < 0.001	0.93 (0.84–0.98) *p* < 0.001	0.86 (0.52–0.99) *p* < 0.001

S_t_O_2_ oxygen saturation; THb total hemoglobin concentration; ICC intra-class correlation coefficient; CI confidence interval.

## Data Availability

Raw data supporting the conclusions of this article will be made available by the authors upon request.
